# Access to Awareness for Faces during Continuous Flash Suppression Is Not Modulated by Affective Knowledge

**DOI:** 10.1371/journal.pone.0150931

**Published:** 2016-04-27

**Authors:** Milena Rabovsky, Timo Stein, Rasha Abdel Rahman

**Affiliations:** 1 Department of Psychology, Humboldt University at Berlin, Berlin, Germany; 2 Department of Psychology, Stanford University, Stanford, California, United States of America; 3 Center for Mind/ Brain Sciences, University of Trento, Trento, Italy; University of Udine, ITALY

## Abstract

It is a controversially debated topic whether stimuli can be analyzed up to the semantic level when they are suppressed from visual awareness during continuous flash suppression (CFS). Here, we investigated whether affective knowledge, i.e., affective biographical information about faces, influences the time it takes for initially invisible faces with neutral expressions to overcome suppression and break into consciousness. To test this, we used negative, positive, and neutral famous faces as well as initially unfamiliar faces, which were associated with negative, positive or neutral biographical information. Affective knowledge influenced ratings of facial expressions, corroborating recent evidence and indicating the success of our affective learning paradigm. Furthermore, we replicated shorter suppression durations for upright than for inverted faces, demonstrating the suitability of our CFS paradigm. However, affective biographical information did not modulate suppression durations for newly learned faces, and even though suppression durations for famous faces were influenced by affective knowledge, these effects did not differ between upright and inverted faces, indicating that they might have been due to low-level visual differences. Thus, we did not obtain unequivocal evidence for genuine influences of affective biographical information on access to visual awareness for faces during CFS.

## Introduction

Affective information about other people acquired without direct experience can greatly benefit social behavior [1]. Recent evidence suggests that such verbally transmitted emotional information can modulate not only how we reason about or act towards another person [2,3], but also how we perceive that person's face [4,5]. For instance, Suess, Rabovsky, and Abdel Rahman [6] showed that associating negative biographical information with previously unfamiliar neutral faces caused participants to judge their facial expressions as more negative. Another recent study suggests that affective information may even have an impact on whether a face is consciously perceived in the first place: Anderson, Siegel, Bliss-Moreau, and Barrett [7] found that faces paired with negative social information (e.g., “threw a chair at his classmates”) dominated longer in visual consciousness during binocular rivalry, a phenomenon which occurs when perceptually incompatible images are presented to both eyes and compete for perceptual dominance [8]. During standard binocular rivalry perception alternates between two competing stimuli such that dominance durations are influenced both by each stimulus’ relative potency to gain access to awareness and to persist in awareness once dominance has been achieved. Here, besides aiming to replicate the influence of affective knowledge on the perception of facial expressions, we specifically focused on the question whether verbally transmitted affective biographical information about faces can modulate their potency to gain initial access to awareness.

We used continuous flash suppression (CFS), a recently developed variant of binocular rivalry, in which a stimulus presented to one eye is suppressed from awareness by flashing dynamic high-contrast pattern masks to the other eye [9]. Potency to gain access to awareness is quantified as the time needed to detect an initially suppressed stimulus [10]. In contrast to standard binocular rivalry, detection under CFS specifically reflects the potency of initially unconscious stimuli to overcome suppression and break into consciousness. Therefore, this “breaking CFS” (b-CFS) technique is often viewed as an indicator of unconscious processing [10,11,12,13,14], but see [15].

It is a controversially debated topic whether access to conscious perception during such interocular suppression is modulated by semantic factors. Some b-CFS studies report evidence suggesting semantic processing of interocularly suppressed stimuli. For instance, Costello, Jiang, Baartmann, McGlennen, and He [16] found that written words (e.g., “fire”) break through suppression faster when they are preceded by a semantically related word (e.g., “burn”) than when they are preceded by an unrelated word. Lupyan and Ward [17] subsequently showed that the presentation of verbal labels facilitates detection across stimulus domains, so that the presentation of the word “pumpkin” induced shorter suppression durations for a subsequently presented image of a pumpkin whereas an unrelated word did not (also see [18,19]). While the advantage of these priming studies is that the same stimuli can be used across the different conditions to avoid differences in low level visual stimulus characteristics, it is not entirely clear whether the target needs to be analyzed semantically in order to facilitate detection and to what extent the activation of the prime and prime-related information feeds back into lower visual areas to bias interocular competition at these early stages. Indeed, Lupyan and Ward [17] suggested that the verbal labels presented as primes activated visual features of the objects they refer to and that the match between this language induced activation with stimulus-driven activity facilitated detection at a perceptual level. However, it is less clear how such perceptual pre-activation can account for the word priming effects obtained by Costello et al. [16].

A number of studies investigated semantic influences on b-CFS by comparing stimulus classes differing in their semantic content including their emotional valence. The advantage of this approach is that any differences between stimulus conditions cannot be attributed to previously induced changes in the neural state including possible changes in perceptual activation. On the other hand, using different stimuli in the different experimental conditions entails the problem that any observed effects may be due to differences in visual stimulus characteristics, which strongly influence detection under CFS [20,21].

For instance, emotional facial expressions have been found to modulate suppression durations during b-CFS [22], but these effects have been shown to be driven by visual factors rather than emotional valence per se [23,24]. Using verbal stimulus material which presumably entails less systematic visual confounds, Yang and Yeh [11] found longer suppression times for words of emotionally negative valence as compared to neutral words. Sklar, Levy, Goldstein, Mandel, Maril, and Hassin [25] compared multi-word expressions of either negative or neutral valence and found verbal expressions with negative valence to break into consciousness faster than neutral expressions. Because the emotional valence was not related to the individual words but resulted from their combination (e.g., ‘electric’ and ‘chair’ forming ‘electric chair’), these results even suggest integration of words during interocular suppression. However, the direction of the effect (shorter suppression times for negative valence) was opposite to the findings reported by Yang and Yeh [11]. Sklar et al. [25] additionally demonstrated that incoherent multi-word expressions (e.g., ‘I drank the chair’) were detected faster than coherent verbal expressions (e.g., ‘I moved the chair’). Similarly, Mudrik et al. [26] found that scenes with incongruent objects (e.g., a woman putting a chessboard in the oven) were perceived faster than the respective congruent versions (e.g., a woman putting food in the oven). In contrast, the above discussed semantic priming effects reported by Costello et al. [16] demonstrate faster detection of targets following semantically congruent primes, and for simple stimuli such as faces or single words, the overall pattern seems to indicate that access to awareness is easier for more typical and familiar stimuli such as faces from the same age and race as the observer [27] or words in a well-known as compared to an unfamiliar alphabet [10]. However, Heyman and Moors [28] did not observe any differences in suppression durations between frequent and rare words, and even between existing and nonexisting words, so that the influence of familiarity on b-CFS does not seem to be ubiquitous.

Overall, the pattern of results seems rather mixed. Specifically, the two studies showing semantic influences of emotional valence on word processing report effects in opposite directions [11,25]. Thus, further evidence concerning high-level emotional influences on b-CFS seems highly desirable. Furthermore, to our knowledge, no evidence is available on semantic influences or emotional valence effects on face processing during b-CFS apart from facial expressions that necessarily include visual manipulations. Here, we used an established affective learning paradigm [4,6] to investigate influences of verbally transmitted affective knowledge on suppression durations of faces with neutral expressions during CFS.

We used both famous faces with emotionally negative, positive, or comparatively neutral biographical information and faces which were initially unfamiliar to participants and which were associated with emotionally negative, positive or comparatively neutral biographical information through an affective learning procedure [4,6]. The assignment of these unfamiliar faces to negative, positive or neutral biographical stories was counterbalanced across participants to control for visual differences between the face stimuli. For the famous faces all participants were provided with the same real biographical information (e.g., negative for Hitler, positive for the Dalai Lama, and comparatively neutral for Ronaldinho), such that full control of visual differences through counterbalancing was not possible. The biographical information provided for the famous faces was intended to refresh the participants’ memory and to increase the credibility of the information provided for the newly learned faces by creating a situation where the participants were led to believe that they saw more or less well-known people, some of which they knew and some of which they did not know. The inclusion of both newly learned and famous faces provides different advantages in the investigation of affective knowledge effects on CFS: Newly learned faces allow for counterbalanced assignment of face stimuli to affective knowledge conditions and thus for perfect control of visual factors. On the other hand, while famous faces necessarily differ between emotion conditions, giving rise to potential visual confounds, they allow investigating influences of affective knowledge acquired through repeated exposure in a variety of real-world contexts that has thus been better consolidated in long-term memory.

Both directly before and after learning, as well as two days later (to examine whether influences of newly acquired knowledge might increase through overnight consolidation), newly learned and well-known faces were presented randomly intermixed during CFS. All faces were presented in upright and inverted orientations, to further control for visual differences. We tested the hypothesis that the potency of upright faces to overcome suppression would be modulated by affective biographical information, as might be expected based on previous evidence [7,11,25]. Furthermore, we obtained facial expression ratings both before and after associating the biographical information, aiming to replicate the influence of affective knowledge on the perception of facial expressions. Due to mixed results obtained in previous studies, we examined, as a side issue, whether there might be inter-individual differences concerning such influences. In general, people differ widely in their responses to social and affective information. To limit the number of analyses and thus the probability of obtaining significant results by chance, we focused on influences of personality (as measured in the NEO Five Factor Inventory) on one specific comparison, namely the difference between neutral and negative affective information associated with newly learned faces, which has been found to affect dominance durations during binocular rivalry [7]. Based on evidence that Neuroticism enhances the processing of negative emotions while Extraversion enhances the processing of positive emotions [29,30], it could be expected that the influence of negative affective information is stronger for participants scoring high in Neuroticism or low in Extraversion. Furthermore, Agreeableness–a measure of the extent to which people are rather suspicious and antagonistic or altruistic towards others and trustful that others will be similarly helpful and benevolent—has been related to emotion regulation in social contexts [30,31], and it might be expected that negative affective information has a stronger impact on participants scoring low in Agreeableness as it matches their suspicious expectations.

To foreshadow our results, we replicated affective knowledge effects on the perception of facial expressions, but did not find evidence for genuine influences of affective biographical information or modulating influences of inter-individual differences on suppression durations during CFS.

## Materials and Methods

### Ethics statement

This research has been conducted according to the principles expressed in the Declaration of Helsinki and was approved by the Ethics Committee at the Department of Psychology, Humboldt University at Berlin. Participants provided written informed consent prior to participation.

### Participants

Forty-eight native German speakers (31 women; mean age = 25, age range 19–34) with normal or corrected-to-normal vision participated in the experiment. They were naïve to the purpose of the study and received either course credit or monetary compensation for their participation.

### Materials

**Fig 1:**
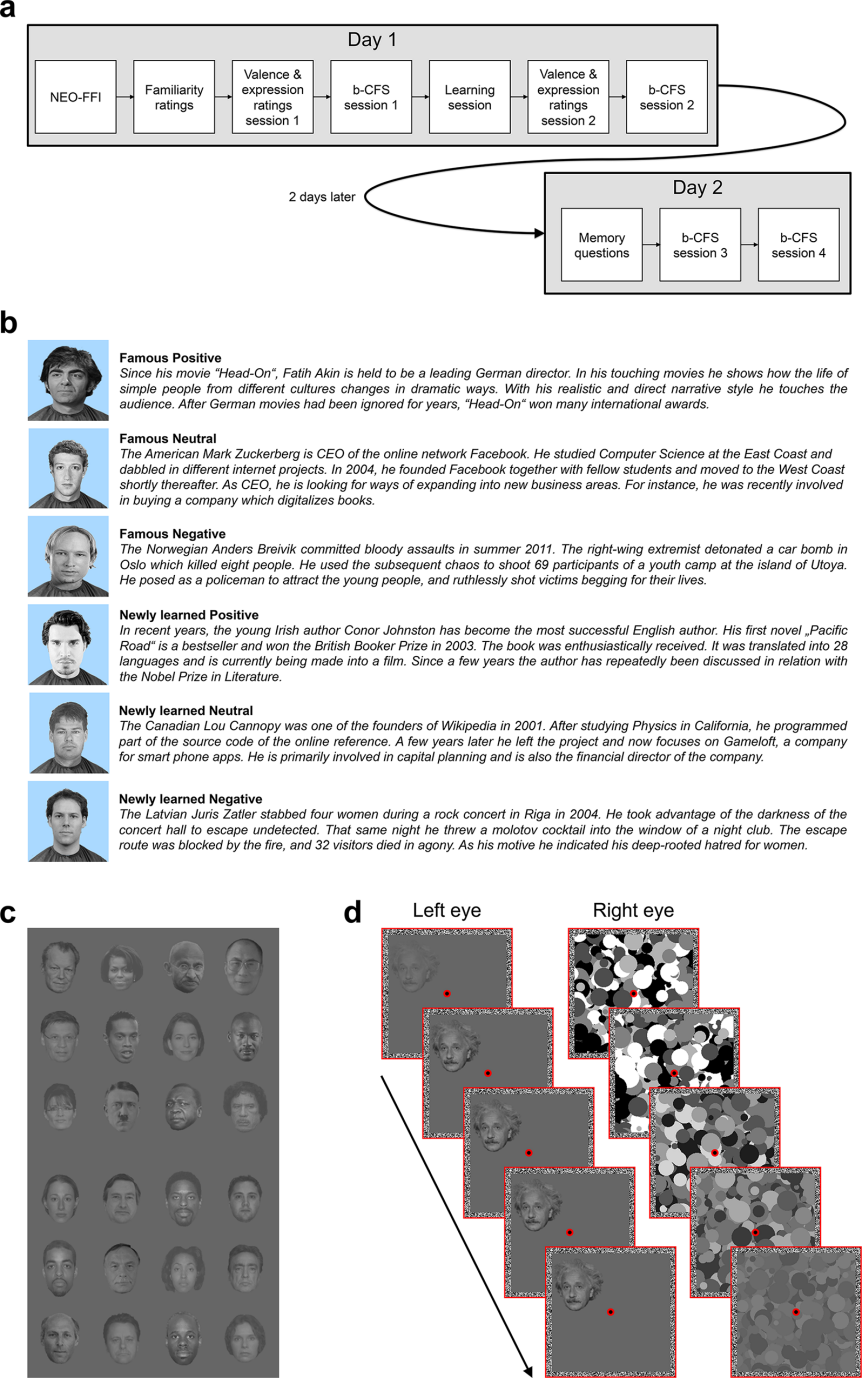
Procedure and stimuli. a) Schematic of the overall procedure. b) Example of a set of famous and newly learned faces with corresponding stories containing affective biographical information. Faces within a set were matched for gender, age, and race, and fictitious stories for newly learned faces were constructed in analogy to the real biographical stories for the famous faces in an attempt to match valence, arousal, etc. Please note that the assignment of faces to stories was fixed for famous faces but counterbalanced for newly learned faces. c) Examples of famous and newly learned faces presented during CFS. d) Schematic of an example b-CFS trial. A face photograph was gradually introduced to one eye. To render the face target invisible for the first seconds of a trial through interocular suppression, CFS masks updated at 10 Hz were presented to the other eye. The contrast of the CFS masks was slowly ramped down over the course of each trial. Participants indicated as quickly and accurately as possible on which side of fixation the target or any part of the target became visible.

Picture stimuli consisted of gray-scale photographs of 36 famous and 36 unfamiliar male and female faces (approximately 3.1° × 4.7°) with neutral facial expressions. Photographs of famous faces were obtained from the internet, while initially unfamiliar neutral faces were taken from various databases including FACES [32] and the Radboud Faces Database [33]. All face stimuli were matched in mean luminance and RMS contrast (based on the monitor’s input values, as we did not linearize the monitor output). We additionally aimed to control for differences in low level stimulus characteristics by including physically identical stimuli shown in inverted orientation (i.e., rotated by 180 degrees) as it is virtually impossible to equate all visual stimulus properties that may influence b-CFS [20,22,24,34]. Thus, the famous face stimuli likely differed along such dimensions, for example in local contrast or spatial frequency content, but importantly, these problems do not apply to the newly learned (initially unfamiliar) faces where the assignment of faces to biographical information was counterbalanced across participants. Spoken stories containing emotionally negative, positive or comparatively neutral biographical information that was either fictitious (for unfamiliar faces) or real (for familiar faces) were recorded and presented auditorily (mean duration ~ 26 s). Stories for the newly learned faces were constructed in analogy to the real biographical stories for the famous faces so that arousal level was similar, and age, race, and gender of the corresponding faces was matched accordingly. In addition, triples of famous faces, pertaining to negative, positive, and neutral conditions, and triples of novel faces which were used for counterbalanced assignment of the analogical stories were also matched in terms of age, gender, and race (please see Fig 1B).

### Procedure

The overall procedure of the experiment is depicted in Fig 1A. At the beginning of the learning session, participants completed the German version of the NEO Five Factor Inventory (NEO-FFI [35]). Subsequently, each person’s face and name were shown on the screen and participants indicated via button press whether or not the depicted persons were familiar to them (1 = known, 2 = unknown). They were told that they should not worry if some of the faces were unfamiliar to them, because some of the depicted people were not very well-known, or were only well-known in their home countries. The familiarity classification supported the categorization of the faces in unfamiliar (*M* = 1.96) and familiar (*M* = 1.60), *F*(1, 47) = 199.58, *p* < .001. Next, participants completed a valence rating of the persons and a facial expression rating for all faces. In the valence rating, they indicated how positive or negative they found each depicted person, and in the facial expression rating they indicated how positive or negative they perceived the facial expression of the depicted person. Both ratings were given on a 5-point-scale ranging from –2 (very negative) to +2 (very positive). Afterwards, the faces were presented on the screen while participants listened to the stories describing biographical details, which were fictitious for the unfamiliar faces and real for the familiar faces. The assignment of the unfamiliar faces to fictitious stories with emotionally negative, positive, or comparatively neutral information was counterbalanced across participants. All stories were presented twice in random order. After learning, there was a second valence rating and facial expression rating.

There were b-CFS sessions both directly before (baseline condition) and after listening to the biographical stories, as well as two subsequent b-CFS sessions two days later which were separated by a short break, and primarily served to examine possible influences of overnight consolidation on affective knowledge effects and to increase the number of trials in order to detect possibly very subtle influences of affective knowledge. At the beginning of the second day, there was a paper and pencil memory questionnaire where each face was depicted and participants were instructed to indicate in keywords what they remembered concerning each person’s biography. On average, they explicitly recalled the central biographical details of 13 of the newly learned faces (range 0 to 27) and of 30 of the famous faces (range 20 to 36).

During the b-CFS sessions, participants viewed the screen dichoptically through a mirror stereoscope. Throughout each session, two fusion frames (10.4° × 10.4°) enclosing a gray background were displayed side-by-side on the screen such that one frame was presented to each eye (see Fig 1D). There was a fixation dot in the center of each frame. In addition to the upright versions of the face photographs, inverted versions were created by flipping the images vertically. High-contrast CFS masks consisting of randomly arranged grayscale circles were generated to induce interocular suppression.

Each b-CFS trial started with a 1 s fixation period. Afterwards, CFS masks (updated every 100 ms) were presented to one randomly selected eye while an upright or inverted face was introduced to the other eye. The faces were gradually faded in over the first second to avoid abrupt transients, and then remained constant until the end of the trial (Fig 1D). They were presented until responding or for maximally 10 s either to the left or to the right of the fixation dot (centered at 2.9°), at jittered vertical positions (centered at maximally 2.4° below or above fixation). Starting 2 s after trial onset the contrast of the CFS masks was linearly reduced from 100% to 0% over a period of 7 s. Participants were asked to indicate at which side of the fixation dot the stimulus emerged from suppression by pressing the left or the right arrow key on the keyboard as fast and as accurately as possible, as soon as any part of the stimulus became visible. There were 24 trials in each condition, i.e., 12 faces, each presented once to each eye during each session. Thus, with the four sessions, there were overall 96 trials in each emotional knowledge condition. Only trials with correct localization responses (M = 98.3%, SD = 2.3%) were included in the analyses.

### Analyses

For the valence and facial expression ratings, we performed repeated measures analyses of variance (rmANOVAs) with factors session (pre vs. post learning), familiarity (newly learned vs. famous) and emotion (positive, neutral, negative). In addition, we correlated valence and facial expression ratings, separately for newly learned and famous faces. For the suppression durations during CFS, we performed a similar rmANOVA with the additional factor inversion (upright vs. inverted) and the factor session including 2 additional levels, namely the first session after overnight consolidation (session 3) and the second session after overnight consolidation (session 4). In general, significant main effects and interactions in the ANOVAs were followed up with lower level ANOVAs. We then performed additional analyses to follow up on the obtained effects for famous faces, namely linear mixed effects analyses to examine the consistency of the effects across individual faces, and a regression analysis to examine contributions of a specific visual factor (spatial frequency content), valence ratings, and familiarity of individual faces to suppression durations. Finally, we performed a regression analysis to examine possible influences of personality variables on emotion effects on CFS.

## Results

### Valence ratings

**Fig 2:**
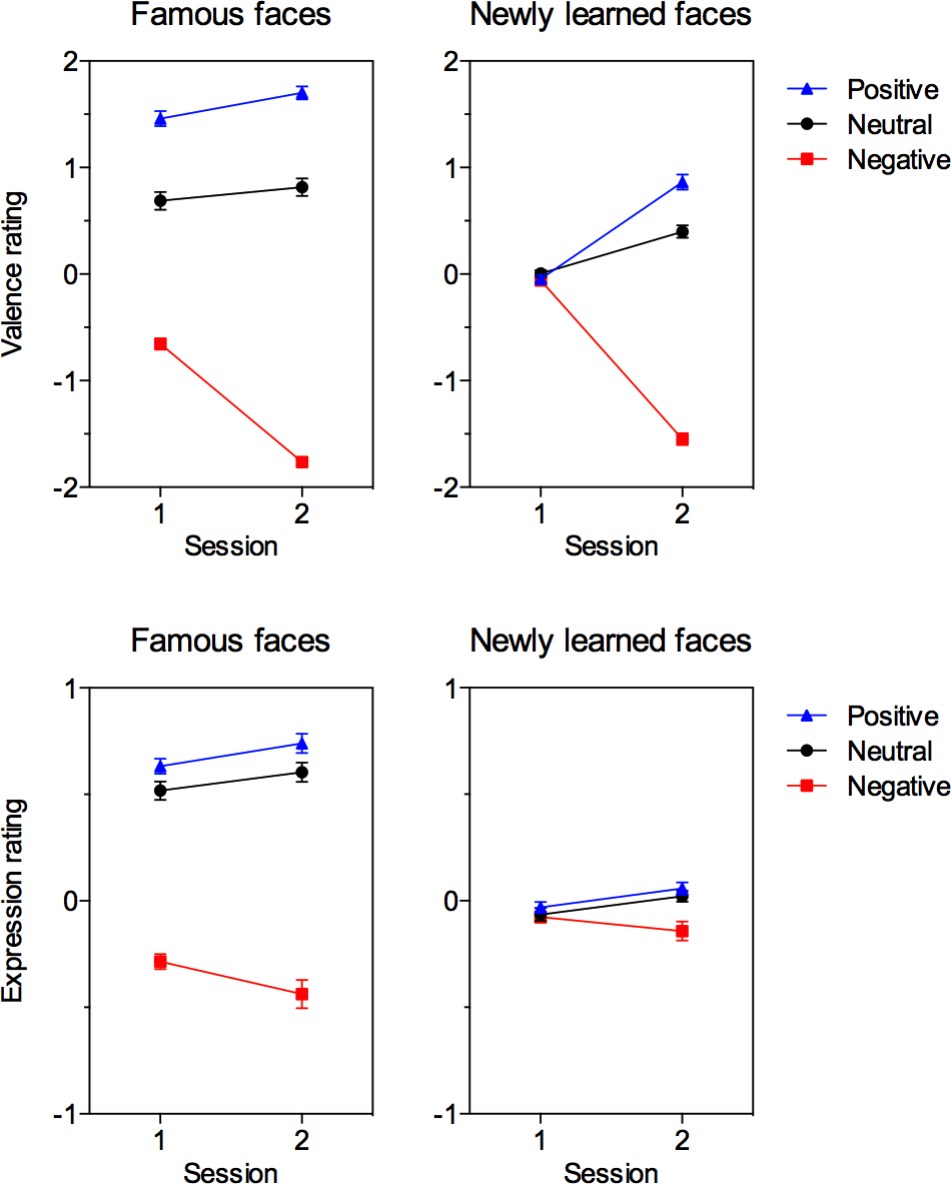
Valence and facial expression ratings. Valence ratings (top) and ratings of facial expressions (bottom) for famous faces (right) and newly learned faces (left). Please note the difference in scaling.

Valence ratings of the persons were employed to control the success of the association of emotional knowledge; the results are displayed in Fig 2, top. It can be seen that for famous faces (left) there were clear differences between affective knowledge conditions in both sessions with only a slight increase of the effects induced by providing the biographical information. On the other hand, for newly learned faces (right) there were no differences between affective knowledge conditions during the first session; differences in valence ratings only developed during the second session after participants were provided with the affective biographical details. A rmANOVA with the factors session (pre vs. post learning), familiarity (newly learned vs. famous) and emotion (positive, neutral, negative) revealed significant main effects of session, *F*(1, 47) = 38.47, *p* < .001, *η*_*p*_*²* = .45, familiarity, *F*(1, 47) = 177.90, *p* < .001, *η*_*p*_*²* = .79, and emotion, *F*(2, 94) = 727.12, *p* < .001, *η*_*p*_*²* = .94, as well as significant interactions between session and familiarity, *F*(1, 47) = 23.67, *p* < .001, *η*_*p*_*²* = .34, between session and emotion, *F*(2, 94) = 670.27, *p* < .001, *η*_*p*_*²* = .93, and between familiarity and emotion, *F*(2, 94) = 213.07, *p* < .001, *η*_*p*_*²* = .82. Finally, there was a significant interaction between session, familiarity, and emotion, *F*(2, 94) = 38.24, *p* < .001, *η*_*p*_*²* = .45.

Post-hoc testing showed that for newly learned faces, the influence of session did not reach significance, *F*(1, 47) = 3.54, *p* = .066, *η*_*p*_*²* = .07, but there was a significant emotion effect, *F*(2, 94) = 366.69, *p* < .001, *η*_*p*_*²* = .89, and a significant interaction between session and emotion, *F*(2, 94) = 389.98, *p* < .001, *η*_*p*_*²* = .89. Before learning, there was no influence of emotion, *F*(2, 94) = 2.42, *p* = .094, *η*_*p*_*²* = .05, but after learning, valence ratings significantly differed according to the associated emotion, *F*(2, 94) = 423.33, *p* < .001, *η*_*p*_*²* = .90, with significantly more positive evaluations of positive as compared to neutral faces, *F*(1, 47) = 70.21, *p <* .*001*, *η*_*p*_*²* = .60, significantly more negative ratings for negative as compared to neutral faces, *F*(1, 47) = 476.07, *p* < .001, *η*_*p*_*²* = .91, and accordingly also a significant difference between positive and negative faces, *F*(1, 47) = 478.23, *p* < .001, *η*_*p*_*²* = .91.

For famous faces, on the other hand, there was a significant influence of session, *F*(1, 47) = 70.34, *p* < .001, *η*_*p*_*²* = .60, a significant influence of emotion, *F*(2, 94) = 622.09, *p* < .001, *η*_*p*_*²* = .93, and also a significant interaction between session and emotion, *F*(2, 94) = 217.54, *p* < .001, *η*_*p*_*²* = .82. Further testing showed that for famous faces, emotion effects were significant both before and after learning, *F*(2, 94) = 251.79, *p* < .001, *η*_*p*_*²* = .84, and *F*(2, 94) = 970.07, *p* < .001, *η*_*p*_*²* = .95, respectively, with significantly more positive ratings for positive as compared to neutral faces, *F*s > 101.50, *p*s < .001, *η*_*p*_*²*s > .67, and more negative ratings for negative as compared to neutral faces, *F*s > 141.98, *p*s < .001, *η*_*p*_*²*s > .74. Thus, also the difference between positive and negative famous faces was significant both before and after hearing the biographical stories, *F*s > 512.06, *p*s < .001, *η*_*p*_*²*s > .91. The interaction between session and emotion was due to a learning-induced change of ratings in opposite directions: While the evaluation of neutral and positive famous faces became more positive after learning, *F*s > 5.10, *p*s < .05, *η*_*p*_*²* > .09, the rating of negative famous faces became even more negative after participants listened to the biographical details, *F*(1, 47) = 410.08, *p* < .001, *η*_*p*_*²* = .90.

### Expression ratings

Influences of affective knowledge on the rating of facial expressions (Fig 2, bottom) were overall smaller than the observed influences on valence ratings (please note the difference in scaling). However, facial expression ratings differed between all affective knowledge conditions for the famous faces (left), and these differences increased through the presentation of the biographical information from session 1 to session 2. On the other hand, for newly learned faces (right), there were no differences during session 1, but during session 2, after providing biographical information, expressions of faces associated with negative information were perceived as more negative than those associated with positive or neutral information. RmANOVAs of facial expression ratings with factors session (pre vs. post learning), familiarity (famous vs. newly learned) and emotion (positive, neutral, negative) revealed significant influences of familiarity, *F*(1, 47) = 256.48, *p* < .001, *η*_*p*_*²* = .85, and emotion, *F*(2, 94) = 148.85, *p* < .001, *η*_*p*_*²* = .76, as well as significant interactions between session and emotion, *F*(2, 94) = 8.19, *p* < .01, *η*_*p*_*²* = .15, and between familiarity and emotion, *F*(2, 94) = 184.45, *p* < .001, *η*_*p*_*²* = .80. Even though there was no significant three-way interaction between session, familiarity, and emotion, we performed a lower level ANOVA looking for interactions between session and emotion separately for newly learned and famous faces, because following the logic and questions of the study it seems to be interesting to examine this interaction separately for familiar and unfamiliar faces. For newly learned faces the rmANOVA with the factors session and emotion revealed a significant influence of emotion, *F*(2, 94) = 6.74, *p* < .01, *η*_*p*_*²* = .13, and a significant interaction between session and emotion, *F*(2, 94) = 5.44, *p* < .01, *η*_*p*_*²* = .10, with further ANOVAs conducted separately for pre- and post-learning showing that the emotion effect for newly learned faces was not significant before learning, *F*(2, 94) = 1.01, *p* = .37, but reached significance after the faces were associated with biographical information, *F*(2, 94) = 8.36, *p* < .001, *η*_*p*_*²* = .15. After learning, facial expressions of persons associated with negative affective knowledge were rated as being more negative as compared to expressions of faces associated with neutral information, *F*(1, 47) = 8.27, *p* < .01, *η*_*p*_*²* = .15, and positive information, *F*(1, 47) = 11.04, *p* < .01, *η*_*p*_*²* = .19, while the difference between neutral and positive faces was not significant, *F*(1, 47) = 1.03, *p* = .32, *η*_*p*_*²* = .022.

For famous faces, the rmANOVA with the factors session and emotion yielded a main effect of emotion, *F*(2, 94) = 223.22, *p* < .001, *η*_*p*_*²* = .83, and an interaction between session and emotion, *F*(2, 94) = 6.63, *p* < .01, *η*_*p*_*²* = .12. The emotion effect was significant both before and after learning, *F*(2, 94) = 213.40, *p* < .001, *η*_*p*_*²* = .82, and *F*(2, 94) = 124.23, *p* < .001, *η*_*p*_*²* = .73, respectively. Follow-up comparisons across both sessions revealed that expressions of negative famous faces were being rated more negative than expressions of neutral famous faces, *F*s > 149, *p*s < .001, *η*_*p*_*²* s > .75, and expressions of positive famous faces, *F*s > 136.75, *p*s < .001, and *η*_*p*_*²* s >.74, and expressions of positive famous faces being rated more positive than neutral famous faces, *Fs* > 7.11, *p*s < .05, *η*_*p*_*²*s > .13. The interaction between session and emotion was due to learning induced changes in opposite directions, as revealed by separate ANOVAs for the three emotions that included the factor session only: While the evaluation of facial expressions of neutral and positive famous faces became more positive from pre- to post-learning, *F*s > 5.69, *p*s < .05, *η*_*p*_*²s >* .*10*, for negative famous faces facial expression ratings became even more negative from pre- to post-learning, *F*(1, 47) = 5.21, *p* < .05, *η*_*p*_*²* = .10.

We also computed correlations between facial expression and valence ratings. For newly learned faces, the correlation was *r*(106) = .482, *p* < .001, before learning, and *r*(106) = .271, *p* < .01 after learning (please note that for newly learned faces, the correlations were computed over instances of specific faces in specific emotional knowledge conditions, thus 36 faces, each in 3 conditions, result in 108 instances). For famous faces, the correlation was *r*(34) =. 537, *p* < .01, before learning, and *r*(34) = .774, *p* < .001, after learning. We suggest that the correlations were most likely driven by the fact that that affective knowledge influenced not only person evaluation but also the perception of facial expressions.

### Breaking continuous flash suppression

**Fig 3:**
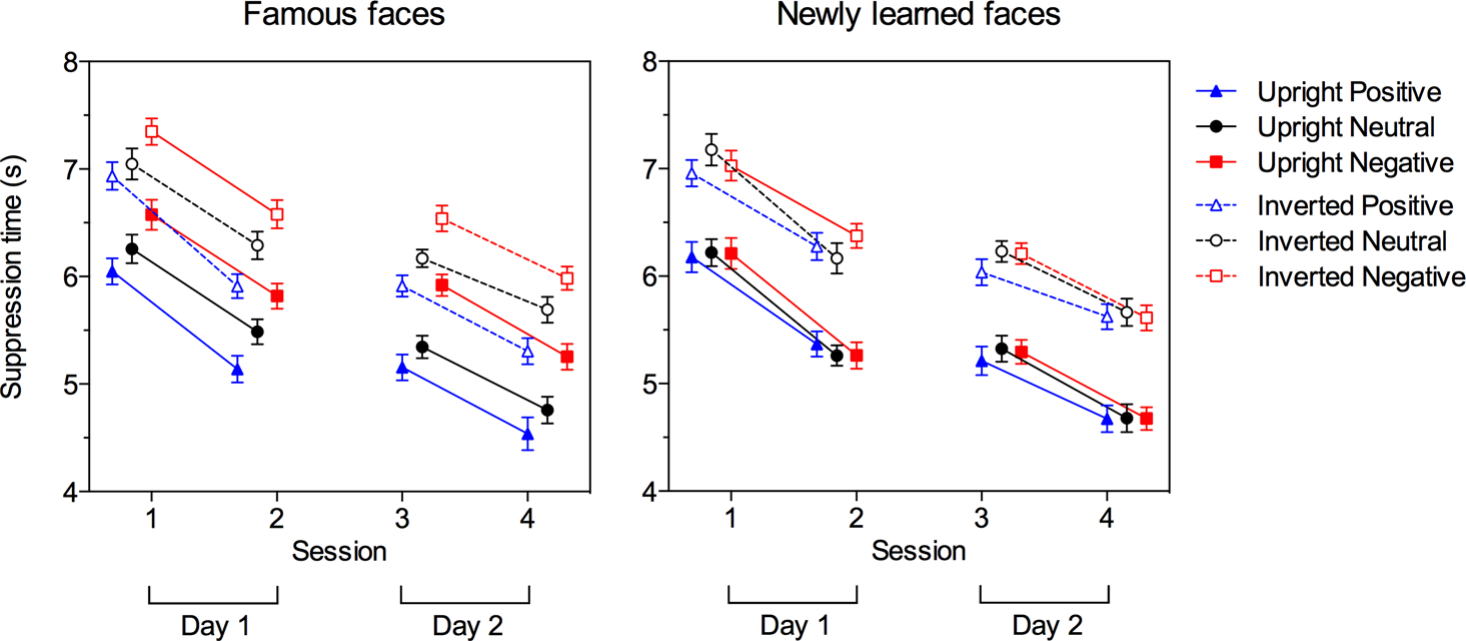
Suppression durations. Mean suppression times for upright and inverted positive, neutral, and negative famous faces (left panel) and newly learned faces (right panel), shown separately for the four separate b-CFS testing sessions. Error bars show SEMs after removal of the irrelevant overall between-subject differences (based on [36]).

Mean suppression durations for famous and newly learned faces associated with positive, neutral and negative biographical knowledge over the four b-CFS sessions are depicted in Fig 3. It can be seen that for famous faces there were clear differences between the emotional knowledge conditions which seemed, however, not to differ between upright and inverted faces. On the other hand, suppression durations for newly learned faces seemed not to be modulated by affective knowledge. Overall, suppression durations were shorter for upright than for inverted faces, and there was a general decrease of suppression durations between subsequent CFS sessions on the same day (sessions 1 and 2 on day 1, and sessions 3 and 4 on day 2). RmANOVAs with factors session (1, 2, 3, 4), familiarity (famous vs. newly learned), inversion (upright vs. inverted) and emotion (positive, neutral, negative) confirmed these visual impressions. They revealed a significant main effect of session, *F*(3, 141) = 26.91, *p* < .001, *η*_*p*_*²* = .36, with decreasing suppression durations between session 1 and 2, *F*(1, 47) = 38.89, *p* < .001, *η*_*p*_*²* = .45, and between session 3 and 4, *F*(1, 47) = 43.52, *p* < .001, *η*_*p*_*²* = .48, but not between session 2 and 3 (*F* < 1). Furthermore, the influence of inversion was highly significant as a main effect, *F*(1, 47) = 168.36, *p* < .001, *η*_*p*_*²* = .78, indicating faster access to consciousness for upright than for inverted faces. There was also a significant influence of emotion, *F*(2, 94) = 58.21, *p* < .001, *η*_*p*_*²* = .55, which was, however, qualified by an interaction between emotion and familiarity, *F*(2, 94) = 44.45, *p* < .001, *η*_*p*_*²* = .49, with no significant emotion effect for newly learned faces, *F*(2, 94) = 1.25, *p* = .29, *η*_*p*_*²* = .03, but only for famous faces, *F*(2, 94) = 71.43, *p* < .001, *η*_*p*_*²* = .60. Importantly, the emotion effect for famous faces did not significantly differ between upright and inverted faces [*F*(2, 94) = 2.27, *p* = .11, *η*_*p*_*²* = .05 for the interaction between emotion and inversion]. In other words, the effect of inversion on suppression durations did not significantly differ as a function of the emotion of the famous faces. Overall, positive famous faces were detected significantly faster than negative famous faces, *F*(1, 47) = 86.03, *p* < .001, *η*_*p*_*²* = .65, and neutral famous faces, *F*(1, 47) = 43.58, *p* < .001, *η*_*p*_*²* = .48, and negative famous faces were detected slower than neutral famous faces, *F*(1, 47) = 60.80, *p* < .001, *η*_*p*_*²* = .56.

Following up on this influence of emotion on suppression durations for famous faces, we explored whether this effect was consistent across individual faces or driven by only a few exemplars. Because we were not interested in differential effects across sessions (famous faces were known to participants before the first session), we first merged the data (only for famous faces) from all four sessions. To account for variability in suppression durations between individual famous faces, we then performed linear mixed effects analyses using the lme4 package [37] for R (R Core Team) on the suppression durations (for details on the analysis approach in the context of b-CFS, see e.g., [27,28,38]). In brief, to test for the main effects of orientation and emotion a reduced model containing random intercepts for participants and for individual face exemplars only was compared against models including additional fixed effects of orientation and emotion, respectively, using likelihood ratio tests to find the model that best fits the data. To test for the interaction effect, a model with the orientation-by-emotion interaction was compared to a model with the two fixed factors only. The comparison of the reduced model with the model containing the additional fixed factor orientation was significant, *χ*^2^(1) = 839.21, *p* < .001, whereas the comparison between the reduced model and the model containing the additional fixed factor of emotion was not significant, *χ*^2^(1) = 2.61, *p* = .11. Although the comparison between the model with the two fixed factors of orientation and emotion with the model containing the orientation-by-emotion interaction did not reach significance, *χ*^2^(1) = 3.43, *p* = .06, to further explore the data we tested the emotion effect separately for upright and inverted faces: Neither for upright famous faces, *χ*^2^(1) = 3.01, *p* = .08, nor for inverted famous faces, *χ*^2^(1) = 2.03, *p* = .16, did the emotion effect reach significance. Thus, these linear mixed effects analyses provided no unequivocal support for an influence of emotion on suppression durations for famous faces.

What other factor then could have given rise to the significant effect of emotion for famous faces that we obtained in the standard analyses of mean suppression durations? Although stimuli were equated for mean luminance and RMS contrast, we did not control spatial frequency content in the images, which is known to influence suppression durations [32,35]. Although low spatial frequencies (LSF) have often been associated with emotional processing [39] previous CFS studies have shown higher spatial frequencies to break through more quickly [34,40]. To test whether differences in spatial frequency content could account for the emotion effect, we computed for every famous face image (i.e., for squares of 136 ×136 pixels encompassing the faces) what proportion of the intensity was coming from a higher-spatial frequency band (more than 4 cycles/image, “HSF-content”). Although mean HSF-content did not differ significantly between positive, neutral, and negative faces, *F* < 1, on average positive famous faces had higher HSF-content (89.6%) than neutral famous faces (89.4%) and negative famous faces (89.3%). Indeed, a correlation analysis revealed a significant negative correlation between suppression durations and HSF-content across individual famous faces, *r*(34) = −.602, *p* < .001, meaning that higher spatial frequency content was associated with shorter suppression durations (a similar correlation between HSF-content and suppression durations was found for newly learned faces, *r*(34) = -.506, *p =* .002). Please note that HSF-content is computed as a proportion so that the LSF-content depends on this estimate. That is, the proportion of the intensity coming from the lower-frequency band (less than 4 cycles/image, LSF-content), is given by [1-HSF-content], and the correlation is therefore the same as for HSF-content, but in the other direction. Changing the cutoff from 4 cycles/image to 5, 3, or 2 cycles/image did not affect these findings. Specifically, the correlation between overall RTs for famous faces and LSF content with a cutoff of 2 cycles/image, *r*(34) = .619, *p* < .001, 3 cycles/image, *r*(34) = .598, *p* < .001, 4 cycles/image, *r*(34) = .602, *p* < .001, 5 cycles/image, *r*(34) = .604, *p* < .001. Thus, LSF-content was consistently correlated with longer RTs, while HSF-content was consistently correlated with faster breakthrough.

A linear multiple regression analysis with valence ratings before learning (which presumably provide a more specific indication of affective knowledge represented in long-term memory than the broad pre-defined experimental categories), familiarity at the start of the experiment, and HSF-content (more than 4 cycles/ image) as predictors, and mean suppression durations for individual famous faces as the dependent variable revealed no significant influences of valence ratings (*β* = -.039, *T* = -.273, *p* = .79) and familiarity (*β* = -.118, *T* = -.830, *p* = .41) but significant decreases in suppression times with higher spatial frequency content (*β* = -.600, *T* = -4.264, *p* < .001). This pattern of results was consistent across variations such as changing the cutoff for the HSF-content, using the valence ratings after learning instead of before learning as predictor, and/ or focusing exclusively on either upright or inverted faces.

Finally, we computed a linear multiple regression analysis with the personality factors measured in the NEO-FFI (Neuroticism, Extraversion, Openness, Agreeableness, Conscientiousness) as predictors and the difference in suppression time between newly learned faces associated with negative as compared to neutral information directly after learning (session 2) as the dependent variable, i.e., a dependent variable corresponding to the condition difference for which [7] observed facilitated perception during binocular rivalry. This analysis showed no influence of any of the personality factors (*β* = -.052, *T* = -.254, *p* = .80 for Neuroticism; *β* = -.120, *T* = -.588, *p* = .56 for Extraversion; *β* = -.139, *T* = -.795, *p* = .43 for Openness; *β* = -.048, *T* = -.274, *p* = .79 for Agreeableness; *β* = -.069, *T* = .417, *p* = .68 for Conscientiousness).

## Discussion

The primary goal of the present study was to investigate the influence of affective biographical knowledge on access to visual awareness for faces during continuous flash suppression (CFS).

Using an established affective learning paradigm [4,6] we associated famous and initially unfamiliar neutral faces with affective biographical information. The assignment of unfamiliar faces to positive, negative, or comparatively neutral biographical information was counterbalanced across participants to control for putative visual differences between face stimuli. Both directly before and after learning, as well as two days later, the faces were presented during CFS, in both upright and inverted orientations to further control for visual influences. In addition, valence and facial expression ratings were obtained before and after the association of biographical information. While replicating affective knowledge effects on the perception of emotional expressions in neutral faces [4,6], we did not find evidence for genuine influences of affective knowledge on suppression durations during CFS.

Specifically, across all CFS sessions, suppression durations for famous faces differed between emotional knowledge conditions, with gradually increasing suppression times from positive over neutral to negative conditions. In contrast, for newly learned faces–where the assignment of faces to emotional knowledge condition was counterbalanced across participants to rule out any influence of putative visual stimulus differences–no effects of affective knowledge were found (see Fig 3). This may suggest that only well-established affective knowledge, but not newly acquired information, influences suppression durations. However, emotion effects for famous faces were very similar for upright and inverted faces. As face recognition is impaired for inverted relative to upright faces [41,42], affective responses may be weakened as well, and genuine influences of emotional knowledge may be reduced by face inversion. The fact that the present emotion effects did not differ significantly between upright and inverted famous faces therefore may point to an influence of uncontrolled visual differences between emotion conditions, which are preserved in inverted faces. Uncontrolled differences between face stimuli are also suggested by the finding that emotion effects failed to reach significance in linear mixed effects analyses with subjects and items as crossed random factors. Further analyses aimed at examining the factors which might underlie the differences between emotion conditions obtained in the standard analyses showed that valence ratings for individual famous faces–which presumably provide a more specific measure of affective knowledge than the broad predefined experimental categories—were not a significant predictor of suppression durations. By contrast, spatial frequency content, a purely low level visual factor, did predict suppression time, with significantly lower durations for higher spatial frequency content. Thus, while it seems conceivable that affective knowledge influences suppression time only after repeated exposure and with long established memory representations—and possibly only after repeated emotional responses [43]–valence ratings had no influence on suppression times whereas high spatial frequency content facilitated detection. However, the interpretation of the differences between affective knowledge conditions in terms of visual confounds is limited by the fact that the visual factor included in our analyses, i.e., spatial frequency content differed only numerically, but not statistically, between the affective knowledge conditions, such that neither valence nor spatial frequency contents can fully account for the observed pattern of results across emotion categories.

We would also like to note that suppression durations did not differ between newly learned and famous faces. Influences of familiarity might have been expected based on previous evidence by Gobbini et al. [44] who observed facilitated perception of familiar faces. However, Gobbini et al. presented personally familiar instead of famous faces which are presumably much more relevant to the participants so that there does not seem to be a direct contradiction between the results. Furthermore, even though the present sample size (*n* = 48) does not allow for reliable conclusions concerning the influence of personality variables, we would like to share the information that a multiple regression analysis showed no relation between any of the five personality factors measured by the NEO-FFI (Neuroticism, Extraversion, Openness, Agreeableness, Conscientiousness) and the influence of negative information on suppression durations for newly learned faces directly after learning (session 2). This dependent measure was chosen because it most closely corresponds to the emotion effect which Anderson et al. [7] observed in dominance durations during binocular rivalry. However, even though Neuroticism and Extraversion have been related to emotion processing [45], and Agreeableness has been linked to emotional regulation in social contexts [31], there was no relation between these variables and the influence of negative affective information on b-CFS.

For newly learned faces that did not show modulated suppression durations, we replicated the influence of affective knowledge on valence judgments and facial expression perception (please see Fig 2). Thus, after learning, faces associated with negative biographical information were judged as more negative and as displaying more negative facial expressions than those associated with neutral or positive information. Besides corroborating previous evidence [4,6], this also demonstrates the success of the present affective learning paradigm. Even though the association of emotion with the faces was explicitly assessed in the present experiment, in an earlier event-related potential (ERP) study using the same stimulus material and learning procedure we observed emotion effects in the ERP which resembled emotion effects observed towards emotional facial expressions during an EEG session two days after providing the emotional information [6]. These results further suggest that the faces indeed elicited emotional processing, also two days after the learning session, so that the lack of evidence for affective knowledge effects on b-CFS cannot be attributed to a failure to associate the emotional information with the face photographs. Furthermore, we replicated the well-established face inversion effect [10], demonstrating that our CFS paradigm was in principle well-suited to reveal differences in suppression times. Thus, concerning the functional localization of affective knowledge effects, the pattern of results suggests that affective information does influence high-level perceptual processes as indicated by influences on the perception of facial expressions, but does not seem to reach a processing level which is sufficiently low-level to be relevant for b-CFS.

The lack of an influence of affective knowledge on suppression durations is in line with the conclusions by Gayet and colleagues [46] who provide a comprehensive review of the complete b-CFS literature published by then, including numerous attempts to show semantic processing during interocular suppression. The authors carefully examine the obtained effects as well as the control conditions used in the different reports of semantic effects on CFS (such as e.g., stimulus inversion, scrambling, or polarity inversion) and conclude that the presently available evidence does not support the view that suppressed stimuli are analyzed semantically. Specifically, four out of five b-CFS studies employing control conditions to dissociate semantic from visual influences attribute the obtained effects to visual factors [23,24,47,48]. On the other hand, two out of four studies attributing their results to conceptual processing of suppressed stimuli during CFS could not conclusively rule out visual accounts of their findings [25,26]. It is important to note, however, that this does not mean that the results necessarily reflected visual differences; it simply means that there were no control conditions to exclude this possibility. Apart from the priming study by Costello et al. [16] (see Introduction), the only study which did include control conditions for visual factors and nevertheless obtained evidence for semantic influences on b-CFS showed faster detection of neutral as compared to negative words [11]. Please note that the polarity of the effect with verbal materials is opposite to what would be expected based on the longer dominance durations of faces associated with negative as compared to neutral social information observed during binocular rivalry [7].

Supporting the conclusions drawn by Gayet et al. [46] and their recommendations to employ proper control conditions, we indeed obtained significant effects of affective biographical information for famous faces, but could not find unequivocal evidence that these effects are not due to visual factors, as they were not reduced by face inversion, failed to reach significance in linear mixed model analyses with subjects and items as crossed random factors, were not obtained in linear regression analyses with valence ratings for individual faces as predictors, and disappeared when using identical face stimuli across affective knowledge conditions, i.e., newly learned faces with counterbalanced assignments of affective biographical details. These results are difficult to reconcile with the view that conceptual factors modulate suppression durations during CFS [16,25,26]. However, future studies should be designed to fully disentangle influences of well-established affective memory representations and visual factors.

In sum, we did not obtain unequivocal evidence for genuine influences of affective biographical information on suppression durations during CFS. These results help clarify the currently controversial role of semantic and emotional factors in the potency of initially invisible stimuli to overcome suppression and break into consciousness during continuous flash suppression.
